# Infective Endocarditis in Patient With Uncorrected Patent Ductus Arteriosus: A Case Report From Rural India

**DOI:** 10.7759/cureus.32004

**Published:** 2022-11-29

**Authors:** Ojas A Mahajan, Gajendra Agrawal, Sourya Acharya, Sunil Kumar

**Affiliations:** 1 Department of Medicine, Jawaharlal Nehru Medical College, Datta Meghe Institute of Medical Sciences, Wardha, IND; 2 Department of Cardiology, Jawaharlal Nehru Medical College, Datta Meghe Institute of Medical Sciences, Wardha, IND

**Keywords:** transthoracic echocardiogram, vegetation, patent ductus arteriosus, congenital heart disease, infective endocarditis

## Abstract

A congenital cardiac defect with an untreated left-to-right shunt is a risk factor for infective endocarditis (IE), particularly right-sided infective endocarditis, which has a distinct clinical presentation and outcomes in comparison to left-sided IE. With a prevalence of at least 2-4 per 1000 term births, patent ductus arteriosus (PDA) accounts for around 10% of all congenital cardiac diseases. Early diagnosis by transthoracic echocardiography and prompt antimicrobial therapy for IE are advised to minimize multiorgan failure and severe pulmonary embolism. Closure of large, hemodynamically significant PDA could have a minimal level of intervention, and it may be done cautiously and efficiently with either surgical or transcatheter procedures. The elimination or minimization of these malformations has been advised to remove or decrease the possibility of IE. We present a case of a 10 years old female who presented with a history of intermittent fevers over two weeks. Clinical examination revealed a PDA murmur. Transthoracic echocardiology (TTE) revealed a PDA with vegetation suggestive of IE. The patient was treated with antibiotics, and two weeks after the antibiotic therapy, a TTE showed resolution of the vegetation. Thereafter, the patient was advised to undergo surgical correction of the PDA. This case report highlights the importance of the association of IE with congenital heart disease.

## Introduction

Infective endocarditis (IE) is an uncommon and life-threatening condition. Its annual incidence ranges from 1.4 to 12.7 cases per 100,000 people. Current developments in the epidemiology of IE in children include a decrease in rheumatic heart disease, increase use of central venous catheters in children with chronic illness, and improved congenital heart disease survival [1]. Infective endocarditis occurs less frequently among children without a previous history of heart disease or other known risk factors [2]. Congenital cardiac disease is a risk factor for developing IE. Infective endocarditis was found in 0.2% to 2% of those with ventricular septal defects (VSD). Compared to people with VSD, the chance of developing IE is lower in patients with patent ductus arteriosus (PDA). The right-sided IE, which can affect either the tricuspid or pulmonary valve, accounts for at least 5% to 10% of all IE cases. Intravenous drug users, patients with congenital cardiac disease, and right-heart instrumentation are all risk factors for right-sided IE [3]. As more sensitive diagnostics modalities, such as Doppler ultrasound, become available, the prevalence of a PDA may increase as previously undetected individuals desire medical support [4]. A large shunt due to PDA can lead to heart failure in the first year of life. About one-fifth of the 240,000 children born with congenital heart disease in India each year would require early intervention to survive through the first year of life [5,6]. Infective endocarditis occurring at the narrow pulmonary end of a PDA is more prevalent in the second decade, and a moderate PDA presenting with heart failure is more common in the third decade. An untreated PDA can progress in developing pulmonary vascular disease, as reflected in Eisenmenger syndrome with shunt flow reversal, ventricular hypertrophy and congestive heart failure, lack of physical development, IE, aneurysmal dilatation of the ductus, and ductal calcification [7].

## Case presentation

Patient information

A 10 years old girl presented to the emergency department of our hospital with intermittent fevers that had persisted for more than two months. She had an episode of fever two weeks before admission. The patient had gone to her primary healthcare and a local hospital, resulting in only minor improvement. The patient had a dry cough, a cold, and a fever on the day of her presentation to our hospital.

Clinical findings

On admission, her blood pressure was 90/60 mm Hg, with a regular heart rate of 130 beats per minute, a respiratory rate of 28 times per minute, and an axillary temperature of 36.5°C. The patient was underweight and pale. Jugular venous pressure was not elevated. A cardiac examination revealed a normal palpable heartbeat and a grade 3/6 continuous machinery murmur at the left first intercostal space (Gibson’s murmur). The findings of lung examination were within normal limits. An abdominal examination revealed no organomegaly. There were no signs of cyanosis or clubbing.

Diagnostic assessment

**Figure 1:**
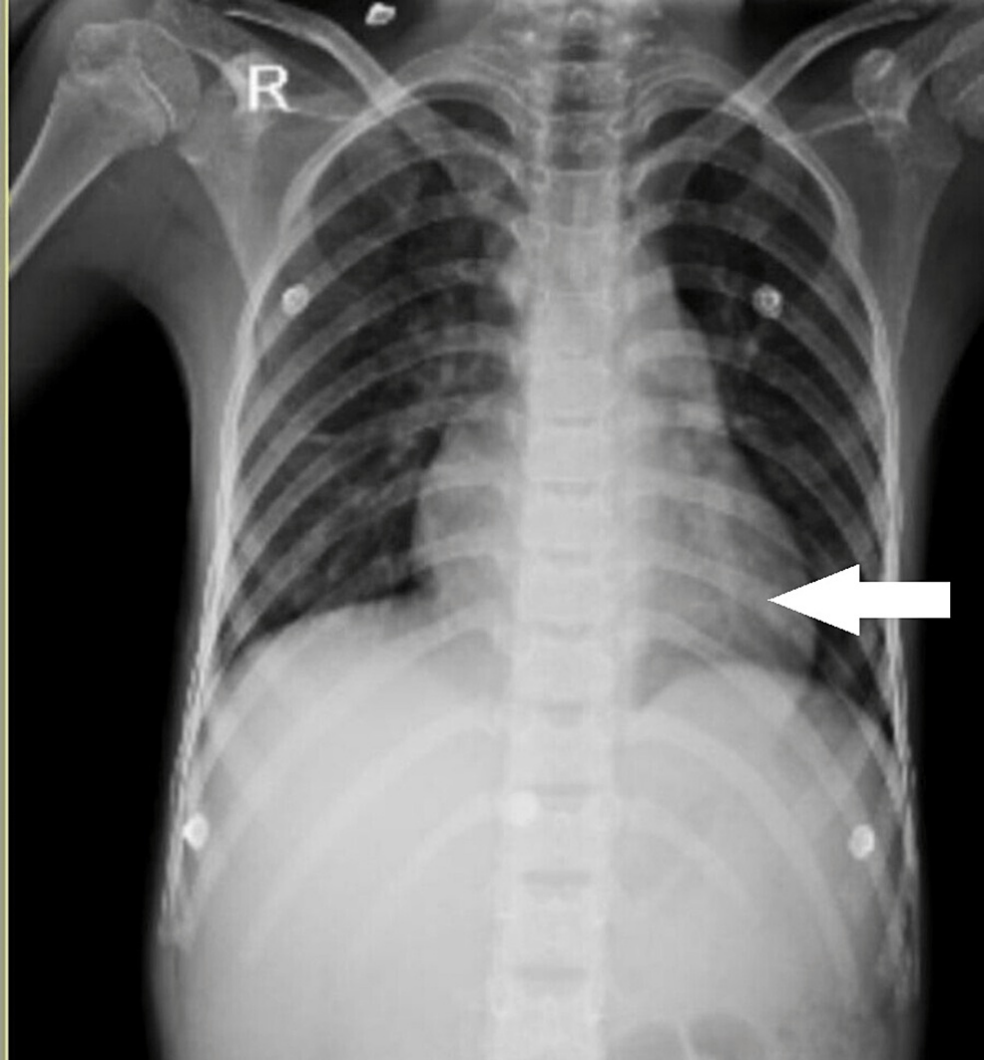
Chest X-ray of the patient revealed cardiomegaly

According to the laboratory results, the hemoglobin level was 12 mg/dL, the leukocyte count was 9200 per cubic mm, and the platelet count was 2,19,000 per cubic mm. The albumin concentration in the blood was 4.4 g/dL. The liver and kidney functions were normal. Chest X-ray revealed cardiomegaly (Figure 1).

**Figure 2:**
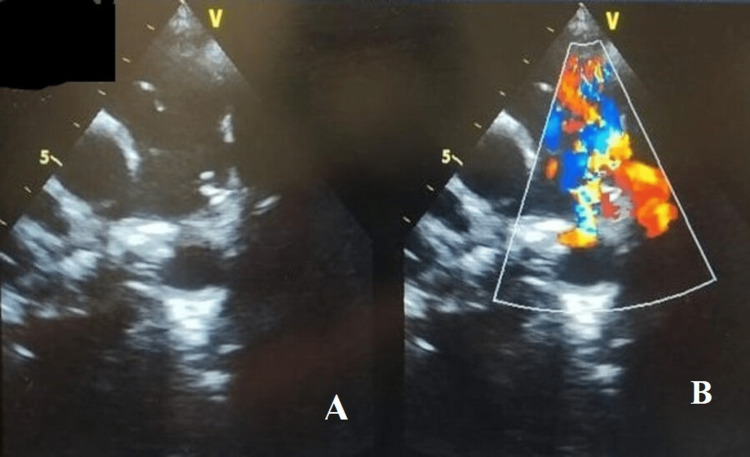
The transthoracic echocardiology (TTE) showed Patent ductus arteriosus (PDA)

TTE revealed a PDA with a left-to-right shunt (Figure 2).

**Figure 3:**
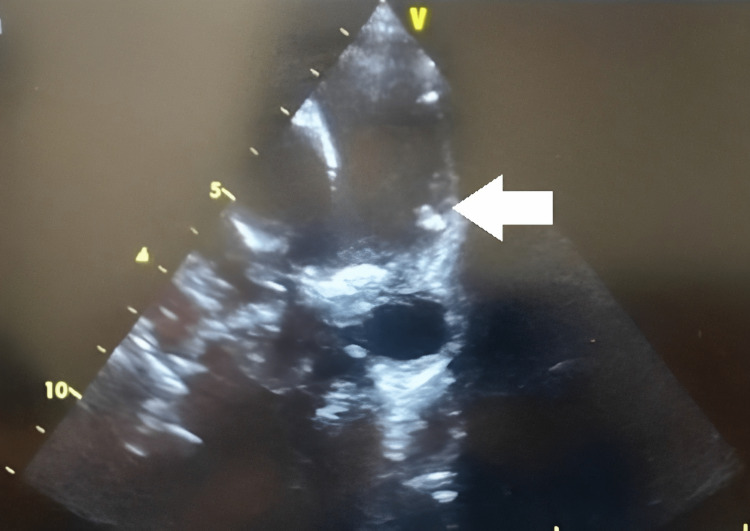
The transthoracic echocardiology (TTE) showed vegetation (white arrow) attached to the pulmonary artery

Vegetation was attached to the pulmonary valve and the main pulmonary artery's wall (Figure 3).

The atrial and ventricular chambers were both dilated. The patient was diagnosed with a left-to-right shunt due to PDA with intermittent fevers, probably due to suspected IE (based on modified Duke criteria). To guide antibiotic treatment, blood samples from three separate sites (right arm, right leg, and left leg) were withdrawn for bacterial culture analysis.

Therapeutic interventions

The patient was administered ampicillin 300 mg intravenously every 24 hours and ceftriaxone intravenously every 12 hours while awaiting blood culture reports. Blood cultures later revealed the growth of *Pseudomonas aeruginosa*. Later the antibiotics were changed to meropenem 800 mg intravenously every eight hours and piperacillin + tazobactam 2 grams every eight hours intravenously, which she received for 14 days.

Follow-up and outcome

After 14 days, the patient had clinically improved and was hemodynamically stable. Repeat TTE showed clearance of the vegetation, and the patient was discharged. The patient was advised to surgical correction of her PDA.

## Discussion

Infective endocarditis (IE) is an uncommon condition that can cause significant morbidity and mortality in children [1]. In recent years, the epidemiology of IE in children has shifted as congenital heart disease (CHD) has replaced rheumatic heart disease as the predominant predisposing factor in developed countries [1,2]. Endocarditis in children has decreased considerably in recent years due to improved dental and medical care for children with congenital heart disease. Although PDA-related endocarditis is uncommon, it is still a risk factor for IE in developing countries [2]. VSD and PDA that have not been corrected are risk factors for IE, particularly right-sided IE.

In our study, the modified Duke criteria for detecting probable IE were fulfilled by exhibiting two main criteria (intracardiac vegetation and positive blood culture) and one minor criterion (predisposing factor of prolonged fevers) [2,3]. The vegetation in the patient's pulmonary artery was the source of turbulent flow in the developed left-to-right shunt. Endothelial cells were damaged by the continuous turbulent flow, which could lead to vegetation formation [3]. *Staphylococcal*, *streptococcal*, and *enterococcal* infections cause 80 to 90 percent of IE cases. In intravenous drug users, *Staphylococcus aureus* is the most common microorganism, whereas, in nondrug users, *Streptococcus viridans* is the most common microorganism [4]. HACEK, a group of fastidious gram-negative microbes, is responsible for a low percentage (5%) of infections (*Haemophilus* species, *Aggregatibacter* species, *Cardiobacterium hominis*, *Eikenella corrodens*, and *Kingella* species). The increased morbidity and mortality caused by gram-negative IE necessitate prompt intervention. Cardiac dysfunction, renal failure, and central nervous system involvement are IE sequelae spurred on by gram-negative microorganisms. Heart failure, annular abscesses, and systemic embolization are generally caused by a pseudomonas infection associated with *E. coli* [5]. More commonly, hospital-based diagnostic and therapeutic procedures, such as venous catheterization for hemodialysis, have seen an increase in the incidence of nosocomial endocarditis. Candida, *Pseudomonas*, *Enterococci*, and *Staphylococci* are the most common organisms identified in cases with intravenous catheter sepsis [6]. One of the rare causes of IE is a malignancy involving atypical sites such as Gingivobuccal Sulcus [7]. 

The PDA's left-to-right shunt enhances pathologic pulmonary vascular shear stress and circumferential stretch by increasing blood flow through the pulmonary artery. The PDA-induced left-to-right shunt increases blood flow from the pulmonary valve into the pulmonary vasculature, causing volume overload in the right ventricle. Increased shear stress and circumferential stretch lead to endothelium breakdown in the turbulence loci, which stimulates the development of vegetation(s) during bacteremia. Fibrin deposition and aggregation are facilitated by endothelial disruption, which leads to vegetation growth [8].

The clinical manifestation of IE has generally been divided into two categories: subacute and acute. Subacute IE is characterized by a low-grade fever that lasts weeks or months and is accompanied by symptoms such as lethargy, chills, myalgia, and weight loss. Acute IE is characterized by a high-grade fever with clinical worsening if not treated promptly. Echocardiography is necessary for the diagnosis and monitoring of vegetational size and heart function. The absence of vegetation on the echocardiogram does not necessarily mean IE is not present [9]. The risk of childhood IE in a large population-based cohort of CHD patients was 6.1/1000 from birth to 18 years of age, or 4.1 cases/10 000 person-years. In childhood, left-sided lesions, cyanotic CHD lesions, and defects in the endocardial cushion had been linked to an elevated risk of IE acquisition [7]. In children under three, the relative chance of having IE was significantly increased throughout the six months following cardiac surgery. The lower frequency of IE in children with CHD than in adults with CHD follows the general population pattern, where the incidence of IE in adults is from 15 to 60 cases per million person-years. However, it is 3.9 to 6.4 cases per million person-years in children [8].

Antibiotics are frequently given intravenously over a long period (4-6 weeks). Candidates for surgical intervention for right-sided IE include severe tricuspid regurgitation with poor treatment response, such as a persistent infection that does not respond to antimicrobial therapy, tricuspid valve vegetation of >20 mm, or recurrent pulmonary embolism. According to most reports, antibiotic treatment for right-sided IE has a reasonable success rate. Surgical intervention is required in 30% of cases [8,9]. Congestive heart failure, valve malfunction, intracardiac abscess, and heart block are all cardiac problems. Sepsis, extracardiac infections (e.g., osteomyelitis and renal abscess), complex immune depositions (e.g., glomerulonephritis), and embolization are examples of extracardiac consequences (e.g., stroke) [9,10].

## Conclusions

This case report represents the dangerous overlap of IE with PDA in which vegetation was attached to the pulmonary artery. The diagnosis of IE with a PDA can raise concerns for future complications of PDA. Our case thus highlights the need for a timely diagnosis and vigilance concerning IE in patients with uncorrected congenital heart disease, including a PDA, for the potential risk of a fatal outcome. 

## References

[1] Sattwika PD, Hartopo AB (2018). Right-sided infective endocarditis in patients with uncorrected ventricular septal defect and patent ductus arteriosus: Two case reports. Clin Case Rep.

[2] Salloum S, Bugnitz CJ (2018). A case report of infective endocarditis in a 10-year-old girl. Clin Pract.

[3] Fortescue EB, Lock JE, Galvin T, McElhinney DB (2010). To close or not to close: the very small patent ductus arteriosus. Congenit Heart Dis.

[4] Saxena A (2005). Congenital heart disease in India: a status report. Indian J Pediatr.

[5] Kamat S, Sagar VV (2022). Escherichia coli urosepsis leading to native valve endocarditis. J Pract Cardiovasc Sci.

[6] Kumar Kumar, Sunil & Diwan, Sanjay (2013) Infective endocarditis due to internal jugular vein haemodialysis catheter. Journal, Indian Academy of Clinical Medicine.

[7] Gagneja S, Shukla S (2020). Infective endocarditis in a pregnant female with chronic sialadenitis associated with gingivobuccal sulcus malignancy: A rare presentation. Jr Cli Diag Res.

[8] Rushani D, Kaufman JS, Ionescu-Ittu R, Mackie AS, Pilote L, Therrien J, Marelli AJ (2013). Infective endocarditis in children with congenital heart disease: cumulative incidence and predictors. Circulation.

[9] Kumar D, Anand P (2016). A case of PDA with infective endocarditis. J Pract Cardiovasc Sci.

[10] Mahmood UF, Durairaj S (2021). Patent ductus arteriosus-related endocarditis: not just a theoretical risk. BMJ Case Rep.

